# Nanoparticle-Enhanced Collagen Hydrogels for Chronic Wound Management

**DOI:** 10.3390/jfb16030091

**Published:** 2025-03-05

**Authors:** Alexandra Cătălina Bîrcă, Mihai Alexandru Minculescu, Adelina-Gabriela Niculescu, Ariana Hudiță, Alina Maria Holban, Adina Alberts, Alexandru Mihai Grumezescu

**Affiliations:** 1 Department of Science and Engineering of Oxide Materials and Nanomaterials, National University of Science and Technology Politehnica Bucharest, 011061 Bucharest, Romania; ada_birca@yahoo.com (A.C.B.); mihaiminculescu27@yahoo.com (M.A.M.); adelina.niculescu@upb.ro (A.-G.N.); agrumezescu@upb.ro (A.M.G.); 2 Research Institute of the University of Bucharest—ICUB, University of Bucharest, 050657 Bucharest, Romania; arianahudita@yahoo.com (A.H.); alina_m_h@yahoo.com (A.M.H.); 3 Faculty of Biology, University of Bucharest, 030018 Bucharest, Romania; 4 Carol Davila University of Medicine and Pharmacy, 050474 Bucharest, Romania

**Keywords:** hydrothermal synthesis, ZnO nanoparticles, CuO nanoparticles, collagen hydrogel, specific antimicrobial properties

## Abstract

Chronic wound infections present a persistent medical challenge; however, advancements in wound dressings and antimicrobial nanomaterials offer promising solutions for improving healing outcomes. This study introduces a hydrothermal synthesis approach for producing zinc oxide (ZnO) and copper oxide (CuO) nanoparticles, subsequently incorporated into PLGA microspheres and embedded within collagen hydrogels. The nanoparticles’ physicochemical properties were characterized using X-ray diffraction (XRD) to confirm crystalline structure, scanning electron microscopy (SEM) for surface morphology, and Fourier-transform infrared spectroscopy (FT-IR) to verify functional groups and successful hydrogel integration. The hydrogels were tested for antimicrobial activity against Staphylococcus aureus, Pseudomonas aeruginosa, and Candida albicans, which are key pathogens in chronic wounds. Biocompatibility was assessed using the human HaCat keratinocyte cell line. Both ZnO- and CuO-loaded hydrogels exhibited broad-spectrum antimicrobial efficacy. Cytocompatibility tests demonstrated that both ZnO- and CuO-loaded hydrogels sustain cell viability and proliferation, highlighting their biocompatibility and suitability for chronic wound healing applications, with superior biological performance of ZnO-loaded hydrogels. Furthermore, the distinct antimicrobial profiles of ZnO and CuO hydrogels suggest their tailored use based on wound microbial composition, with CuO hydrogels excelling in antibacterial applications and ZnO hydrogels showing potential for antifungal treatments. These results underscore the potential of nanoparticle-based collagen hydrogels as innovative therapeutic tools for managing chronic wounds.

## 1. Introduction

Dressings represent the most effective and widely accessible solution for patients with acute and chronic wounds. Applying protective material over wounds has been standard practice for many years. Today, modern dressings not only cover wounds but also play a crucial role in promoting the healing process [1,2,3]. Dressings must meet specific criteria tailored to the unique needs of acute or chronic wounds in order to optimize wound healing. An ideal dressing should possess several essential properties, including the ability to maintain a moist wound environment to facilitate healing, permeability to allow for gas exchange [4], effective management of excess exudate through absorption, robust protection against a wide range of infectious microorganisms [5], adequate mechanical strength, user-friendly features for easy application and removal, biocompatibility and biodegradability [6], mitigation of discomfort and pain, and cost-effectiveness for the patient. By adhering to these guidelines, dressings can significantly enhance the healing process and reduce patient recovery time [7,8,9].

In addition to the primary classification of wounds into two categories—acute and chronic—wounds can be further categorized based on several factors, including the skin’s integrity, the underlying cause, depth, and severity [10]. This classification system encompasses a variety of wound types, such as dry or moist wounds, hot or cold burns, and superficial wounds, as well as those that are partially superficial, partially deep, or full thickness. By understanding these distinctions, healthcare providers can better assess and treat wounds according to their specific characteristics and needs [11,12]. The various characteristics of the wounds significantly influence the healing process. In generally healthy individuals, wound healing typically progresses through four stages: hemostasis, inflammation, proliferation, and remodeling. However, in the case of chronic wounds, the natural healing trajectory often becomes disrupted, with the wounds becoming stuck in the inflammatory stage. Chronic wounds are often exacerbated by underlying conditions such as diabetes, peripheral artery disease, and immunosuppression, which impede the body’s natural ability to regenerate and heal. As a result, understanding these influences is crucial for developing effective treatment strategies for acute and chronic wounds [13,14,15]. Based on these findings, wounds may heal within the typical and required timeframe for an acute wound or persist for extended periods, transitioning into a chronic state [16].

Chronic wounds are often complicated by infections, which can prolong the healing process and pose significant challenges if not addressed from multiple angles. One of the most effective strategies for managing these wounds is specialized dressings. Beyond promoting skin regeneration, these dressings provide targeted protection against infection in the affected area. Maintaining a moist environment with appropriate dressings accelerates healing by preventing dehydration and promoting essential physiological responses, such as angiogenesis and collagen production. Therefore, selecting proper dressings is crucial for improving outcomes in managing chronic wounds [17,18]. Of the various types of modern dressings available—such as foams, hydrocolloids, films, and hydrogels—hydrogels are among the most highly regarded for their effectiveness, adaptability, and potential for improvement. These dressings are extensively utilized in soft tissue engineering applications, including skin, blood vessels, and muscle repair [19]. Hydrogels are characterized by their three-dimensional networks that possess exceptional exudate absorption capabilities while allowing optimal oxygen diffusion [20]. These properties are critical in accelerating wound healing, making hydrogels a top choice for managing chronic and acute wounds [21,22,23]. The choice of suitable material for creating dressings is also an important factor. Due to the complications already present in skin wounds, it is desired to use a dressing that does not induce other possible undesirable effects, so it is of interest to use a dressing made from natural materials. Collagen stands out as one of the most used natural materials in dressing development due to its excellent biocompatibility, impressive fluid absorption capacity, and ability to support cell proliferation. Collagen-based dressings can be engineered as porous scaffolds, facilitating controlled oxygen diffusion to the wound and promoting cell migration and adhesion to the affected tissue. Additionally, collagen closely resembles the natural collagen found in the extracellular matrix, which plays a role in the management of the inflammatory response and the release of growth factors and remodeling of the extracellular matrix. These key properties of collagen not only enhance the effectiveness of wound dressings but also promise improved wound healing outcomes [24,25,26,27,28,29,30].

The ability to tailor treatments to the specific needs of wounds has led to innovative solutions incorporating nanoparticles with high antimicrobial efficacy. These advanced materials are designed to combat chronic wounds that have not healed due to the presence of bacterial colonies. By leveraging the unique properties of nanoparticles, we can enhance wound healing through targeted antimicrobial action, addressing one of the critical barriers to recovery [23,31]. There are various strategies for creating hydrogels with antimicrobial properties. These techniques for producing antimicrobial hydrogels involve three key approaches: the incorporation of metal nanoparticles with inherent antimicrobial properties (e.g., silver, zinc oxide, copper oxide, magnetite, gold nanoparticles, etc.), the addition of antibiotic-type substances (e.g., penicillin, gentamicin, methicillin, vancomycin, ciprofloxacin, etc.), and the use of polymeric materials that also possess natural antimicrobial characteristics as a foundational matrix (e.g., chitosan, alginate, hyaluronic acid, etc.) [32,33,34,35]. The addition of certain specific essential oils (such as rosemary, eucalyptus, lavender, and cinnamon, among others) is another way to provide an antimicrobial effect to hydrogels [36,37]. The incorporation of nanoparticles is likely the most effective approach due to their advantageous characteristics. Silver and zinc oxide are among the most widely recognized and utilized nanoparticles, while other metallic nanoparticles such as gold, copper oxide, magnetite, cerium dioxide, titanium dioxide, calcium peroxide, and magnesium oxide have also been explored and evaluated in scientific papers. These nanoparticles offer diverse possibilities for enhancing the antimicrobial effectiveness of hydrogels in wound treatment applications [35,38,39,40,41,42]. The physicochemical properties of nanoparticles are directly linked to their antimicrobial activity and efficacy. Consequently, the development of nanoparticles for these applications must be approached with meticulous precision and clearly defined objectives. This careful tailoring is essential to ensure that the resulting particles possess the necessary characteristics to effectively combat infections [43,44].

Therefore, integrating nanoparticles with polymer matrices represents one of the most practical and effective solutions to create hydrogels with regenerative and antimicrobial properties for treating chronic wounds [45,46,47]. In this study, we aimed to develop a collagen-based hydrogel dressing, recognized for its exceptional properties for skin application, incorporating two distinct types of nanoparticles: zinc oxide and copper oxide. These nanoparticles were integrated into poly(lactic-co-glycolic) acid (PLGA) microspheres to facilitate controlled release and enhance the synergy with the collagen matrix. As a result, we produced two types of hydrogels: one containing zinc oxide nanoparticles within the polymeric microspheres and another with copper oxide nanoparticles. This research focuses on evaluating and comparing the physicochemical and biological properties of these hydrogels to identify an effective solution for the healing of chronic wounds.

## 2. Materials and Methods

### 2.1. Materials

To develop hydrogels containing polymeric spheres and nanoparticles that are resistant to microbial colonization, the materials listed in Table 1 were necessary. Sigma-Aldrich (Darmstadt, Germany) supplied all substances used, which were of analytical-grade purity.

### 2.2. Zinc Oxide (ZnO) Nanoparticle Synthesis

Zinc oxide nanoparticles were prepared using the hydrothermal synthesis method. Initially, a zinc precursor solution was prepared by dissolving zinc nitrate hexahydrate in methanol at a concentration of 84 mM within a total volume of 200 mL. Next, a 2% sodium hydroxide solution was prepared in water to facilitate precipitation. The sodium hydroxide solution was gradually added to the zinc precursor solution, during which a noticeable color change to milky white indicated the formation of a zinc hydroxide precipitate. To convert the precipitate into zinc oxide nanoparticles, it was transferred to the SynthWAVE (Milestone SRL, Sorisole BG, Italy) synthesis equipment with the following parameters: a temperature of 160 °C, a pressure of 10 bar (ramping from 5 bar to a maximum of 10 bar), and a duration of 20 min. After the thermal synthesis, the resulting product was washed five times with ultrapure water, with centrifugation at 6000 RPM for 10 min after each wash. The final powder was then dried at 40 °C for 16 h and ground vigorously to yield a fine powder of zinc oxide nanoparticles.

### 2.3. Copper Oxide (CuO) Nanoparticle Synthesis

We employed the same hydrothermal synthesis method to enable a comparative analysis of the nanoparticles’ activity to produce copper oxide nanoparticles. The procedure commenced with preparing a 0.1 M solution of copper(II) nitrate hemipentahydrate, dissolved in 100 mL of water. A 4% sodium hydroxide solution was then prepared in a total volume of 200 mL of water and added dropwise to the copper precursor solution. This addition resulted in a noticeable color shift from light blue to dark blue. The subsequent step involved hydrothermal treatment of the solution using the SynthWAVE (Milestone SRL, Sorisole BG, Italy) equipment. The synthesis parameters were consistent with those used for the zinc oxide nanoparticles: a temperature of 160 °C, a pressure of 10 bar (ramping from 5 bar to a maximum of 10 bar), and a duration of 1 h. Following this thermal treatment, the solution turned black, indicating the successful formation of copper oxide nanoparticles. The resulting solution was then washed three times using centrifugation with ultrapure water. After washing, the product was dried in an oven at 40 °C for 16 h. Once dried, the material was ground to yield a fine powder of copper oxide nanoparticles.

### 2.4. PLGA Microsphere Formation

The polymer microspheres were synthesized using the microemulsion method. For this process, 10 mL of a 1% polyvinyl alcohol (PVA) hydrophilic solution and 10 mL of a 0.01% poly(lactic-co-glycolic acid) (PLGA) hydrophobic solution were individually homogenized before being combined. The resulting PVA-PLGA mixture was subjected to ultrasonication with a probe set to an amplitude of 40%. The sonication lasted for 5 min, alternating between 5 s of sonication and 3 s of pause to facilitate the formation of the microspheres. After ultrasonication, the mixture was continuously stirred for 8 h to allow for the evaporation of chloroform. The microspheres were then washed and centrifuged at 6000 RPM for 10 min, resulting in their dispersion in 2 mL of water for subsequent applications. The same technological process was employed to incorporate nanoparticles into the PLGA polymeric microspheres, with the only difference being the addition of 10 mg of each nanoparticle powder (zinc oxide and copper oxide) into the hydrophilic PVA solution. This process resulted in the formation of three distinct samples of PLGA polymeric microspheres: control microspheres without nanoparticles (Mctrl), microspheres containing zinc oxide nanoparticles (MZnO), and microspheres containing copper oxide nanoparticles (MCuO).

### 2.5. Hydrogel Formation

**Figure 1:**
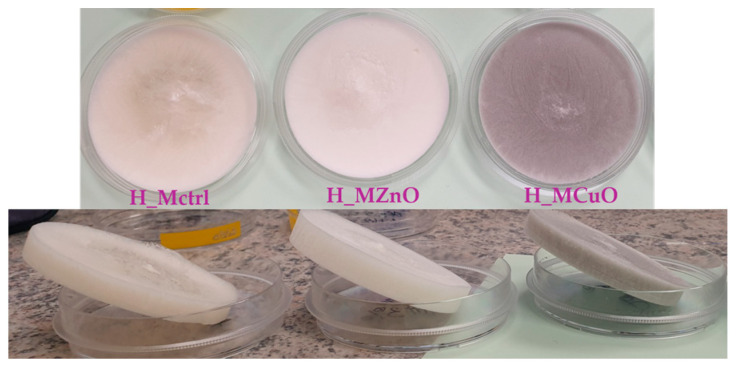
Macroscopic aspect of the obtained hydrogels.

The hydrogels were formed using 200 mg of dry collagen dissolved in a 2% acetic acid solution, resulting in three distinct samples. The first sample, containing collagen only, was designated as the control sample (H_ctrl). The second sample included MZnO in the collagen matrix and was labeled as H_ZnO. The third sample included MCuO within the collagen matrix, and it was designated as H_CuO. To induce crosslinking, the collagen gels were treated with a 1% glutaraldehyde solution and then placed in the refrigerator for 24 h to allow crosslinking. Afterward, the gels were thoroughly washed with ultrapure water to remove residual glutaraldehyde. The final steps involved freezing the gels at −20 °C for 12 h, followed by lyophilization for 72 h to obtain the final hydrogel products (Figure 1). For ease of reference throughout the manuscript, Table 2 summarizes the codes assigned to each sample produced in this study.

### 2.6. Physicochemical Characterization

#### 2.6.1. X-Ray Diffraction (XRD)

The samples of zinc oxide and copper oxide powder were analyzed using a PANalytical Empyrean (PANalytical, Almelo, The Netherlands) model diffractometer purchased from PANalytical in Almelo, The Netherlands. This analysis aimed to investigate the samples’ phase composition, crystallinity, and crystal parameters. The equipment features a hybrid monochromator (2xGe 220) on the incident side, while the diffracted side is equipped with a parallel plate collimator attached to a PIXcel 3D detector. Measurements were conducted using Grazing Incidence X-ray Diffraction (GIXRD) at room temperature, with an angle of incidence of ω = 0.5° and Bragg angles (2θ) ranging from 10° to 80°. The analysis utilized Cu Kα radiation with a wavelength of λ = 1.5406 Å, operating at 40 mA and 45 kV.

#### 2.6.2. Scanning Electron Microscopy (SEM)

SEM analysis was conducted on all obtained samples due to its ability to provide detailed information about the morphology and size of the nanoparticles, polymeric microspheres, and hydrogels. Relevant data were gathered using the FEI Quanta Inspect F50 electron microscope from Thermo Fisher Scientific (Hillsboro, OR, USA). The high vacuum method was employed for sample investigation, utilizing secondary electron mode at 30 keV. Additionally, the SEM equipment is equipped with an energy dispersive spectroscopy (EDS) module, which allows for the identification of the elemental composition of the samples.

#### 2.6.3. Fourier Transform Infrared Spectroscopy (FTIR)

All samples underwent FTIR analysis to identify their chemical composition by detecting specific functional groups at designated wavenumbers. A ZnSe crystal was utilized in the Ni-COLET 6700 FT-IR spectrometer, acquired from Thermo Fisher Scientific (Madison, WI, USA), to conduct the FTIR analysis. Spectra were collected after 64 scans across a wavenumber range of 4000 to 400 cm^−1^, with a resolution of 4 cm^−1^. The resulting data were processed using the OmnicPicta software (version 8.2, Thermo Fisher Scientific, Madison, WI, USA).

#### 2.6.4. Dynamic Light Scattering (DLS)

DLS analysis offers critical insights into the hydrodynamic diameter and zeta potential of the synthesized materials. To obtain accurate measurements, we utilized a DelsaMax Pro from Beckman Coulter (Brea, CA, USA). Both nanoparticles and microspheres were dispersed in distilled water in a 1:20 (w:v) ratio, with the same ratio applied for the microspheres. The samples were then subjected to ultrasonication for 10 min to ensure uniform dispersion. Following preparation, the samples were injected into the measurement cell, and triplicate measurements were conducted to ensure reliability.

#### 2.6.5. Swelling Rate

The hydrogels were shaped into round disks with a diameter of 5 mm using a bio punch. Each sample was assessed for its swelling capacity by measuring the increase in mass upon contact with a phosphate-buffer solution (PBS). The swelling rate was determined using the following formula:

$$Swellingratio=\frac{Wt-Wi}{Wi}\times 100\%$$

where *Wi* represents the mass of the dry sample (before PBS immersing) and *Wt* is the mass of the swelled hydrogel at specific points in time.

#### 2.6.6. Degradation Rate

The degradation rate of the hydrogel was assessed following the swelling rate evaluation, specifically after a 24-h period. In this context, the initial stage of degradation was estimated using the following formula:

$$Degradation=(1-\frac{Wi-Wt}{Wi})\times 100\%$$

where *Wi* denotes the mass of the dry sample (prior to immersion in PBS), and *Wt* represents the mass of the hydrogel after 24 h of immersion in PBS.

### 2.7. Biofilm Evaluation

To evaluate the effect of the obtained dressings on biofilm production, the materials were cut into 1 cm x 1 cm squares and sterilized by UV radiation for 20 min on each side. A sterile fragment of the material was then placed in a well of a sterile 6-well plate. Following this, 2 mL of simple broth liquid medium was added to each well, along with 50 μL of microbial suspension at a 0.5 McFarland density. The prepared 6-well plates were incubated at 37 °C for 24 h. After incubation, the materials were washed with AFS, and the medium was replaced to promote further biofilm development. The plates were incubated for an additional 24 h. At the end of each incubation period, the samples on which biofilms had formed were washed with AFS and transferred to a sterile tube containing 1 mL of AFS. The tube was then vortexed vigorously for 30 s to dislodge the cells from the biofilm. The resulting cell suspension was diluted, and various dilutions were plated on a solidified culture medium to quantify the colony-forming units (CFU/mL).

### 2.8. Biocompatibility Evaluation

To investigate the biocompatibility of the materials, the HaCaT keratinocyte cell line was employed. The cell line was cultured in Dulbecco’s Modified Eagle’s Medium (DMEM) supplemented with 10% fetal bovine serum (FBS) and 1% antibiotics mix throughout the experiments. The cells were maintained in standard cell culture conditions (5% CO_2_, 37 °C). Prior to the biological investigations, the materials were sterilized by UV exposure and transferred to 12-well sterile plates. HaCaT cells were seeded onto the material surfaces at an initial density of 1 × 10^4^ cells/cm^2^ and incubated for 24 h and 72 h under standard culture conditions. The following cell parameters were assessed as indicators of cell health to investigate the biocompatibility of the novel materials: (i) cell viability and proliferation potential, (ii) materials cytotoxicity, and iii) cell morphology.

To investigate cell viability and proliferation potential, MTT assay was employed. After each time point, the culture medium was discarded and the materials were incubated with a fresh solution of MTT (3-(4,5-Dimethylthiazol-2-yl)-2,5-Diphenyltetrazolium Bromide) (Sigma Aldrich, 1 mg/mL in serum-free DMEM, 4 h, 37 °C). After 4 h, the resulting formazan crystals were solubilized in DMSO, and the absorbance was measured at 550 nm using the multimode microplate FlexStation III (Molecular Devices, San Jose, CA, USA). The results are presented as % of cell viability considering the mean optical density (O.D.) obtained for the experimental control at 24 h as 100% of cell viability.

The release of lactate dehydrogenase (LDH) was quantified using the In Vitro Toxicology Assay Kit TOX7, Lactic Dehydrogenase based (Sigma Aldrich) according to the manufacturer’s protocol. Briefly, 50 µL of supernatant was collected from each well at the designated time points and mixed with 100 µL of the reaction solution. After incubation for 30 min at room temperature in the dark, absorbance was measured at 490 nm at the multimode microplate FlexStation III.

Actin cytoskeleton organization was assessed by fluorescence microscopy after staining with phalloidin-TRITC. Following the removal of the culture medium, cells were fixed in 4% paraformaldehyde (20 min, RT) and permeabilized with 0.1% Triton X-100/2% bovine serum albumin (BSA) (30 min, RT). Cells were then incubated with phalloidin-TRITC (1:100 in PBS, 1 h, 37 °C, in the dark), washed with PBS, and counterstained with DAPI to visualize nuclei. Stained samples were examined using the Olympus IX73 fluorescent microscope (Olympus Life Science, Waltham, MA, USA) and CellSense F software v8.0.2.

## 3. Results and Discussion

### 3.1. Characterization of Powder-Type Samples: ZnO and CuO

**Figure 2:**
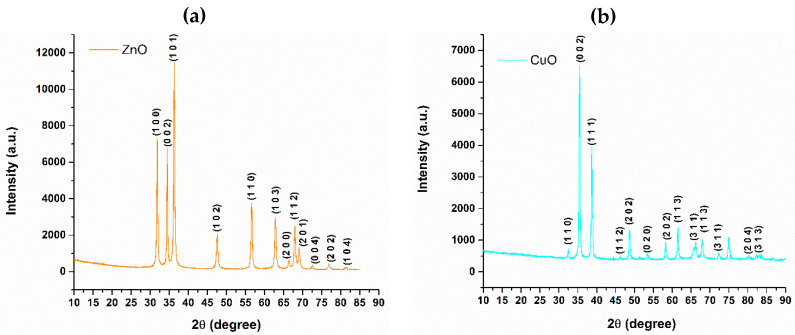
XRD analysis results for (**a**) ZnO and (**b**) CuO powder samples.

XRD analysis was conducted to characterize the zinc oxide and copper oxide nanoparticle samples in terms of their crystallinity and component phases. This characterization is crucial for predicting their properties, particularly in relation to their potential use in combating microbial infections. Figure 2 displays the diffractograms obtained from the XRD analysis of the zinc oxide and copper oxide powders.

Upon analyzing the diffractograms, it is evident that each oxide powder exhibits a single crystalline phase, confirming the crystalline nature of both samples. In the diffractogram for zinc oxide, distinct diffraction maxima are observed at 2θ angles of 31.803°, 34.455°, 36.301°, 47.585°, 56.633°, 62.873°, and 67.995°, characteristic for zinc oxide crystal structure. According to the information from the PDF-ICDD database, these diffraction maxima correspond to the following principal crystal planes: (100), (022), (101), (102), (110), (103), and (112). Additionally, the crystallite size was calculated using the Debye-Scherrer equation, having a value of 24.79 nm.

Similar information was observed in the diffractogram of the copper oxide sample. Diffraction peaks are observed at 2θ angles of 35.477°, 38.727°, 48.711°, 58.253°, 61.529°, 67.951°, and 74.971°, being indicative of the copper oxide crystal structure, based on the information from the PDF-ICDD database. The identified crystal planes ((002), (111), (−202), (202), (−113), (113), and (004)) were in concordance with the aim of obtaining copper oxide nanoparticles. The calculated crystallite size using the same Debye–Scherrer equation was 40.95 nm.

**Figure 3:**
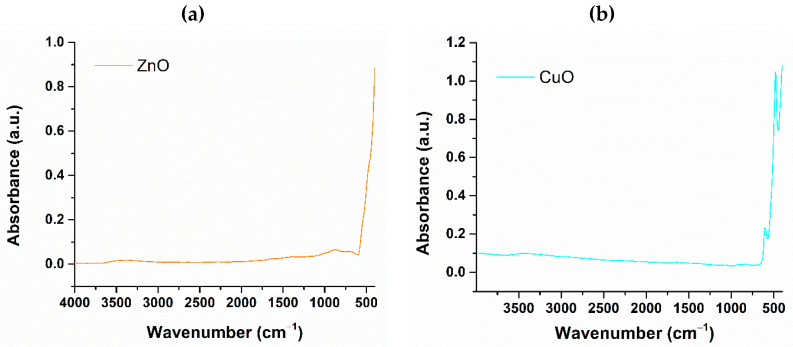
FTIR spectra of synthesized (**a**) ZnO and (**b**) CuO nanoparticles.

To obtain additional information on the metal oxide powders, FTIR analysis was conducted to identify the functional groups associated with their chemical structures. Figure 3 displays the FTIR spectra obtained for each of the two nanoparticle powders individually.

For both nanoparticle samples, absorption bands are observed at wavenumbers indicative of oxygen/metal bonds below 1000 cm^−1^. Specifically, zinc oxide exhibits an absorption band around 401 cm^−1^, which corresponds to the zinc/oxygen bond. In contrast, the FTIR spectrum of the CuO nanoparticle sample reveals the presence of two vibrational bands at 601 cm^−1^ and 479 cm^−1^, both associated with the copper/oxygen bond. Thus, the FTIR spectroscopy results further validate the successful synthesis of zinc oxide and copper oxide nanoparticles, respectively.

**Figure 4:**
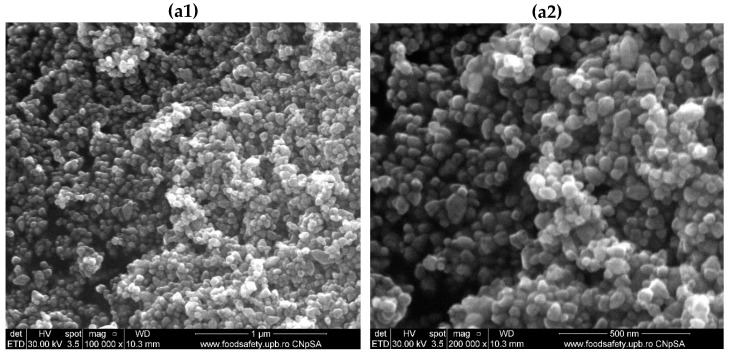

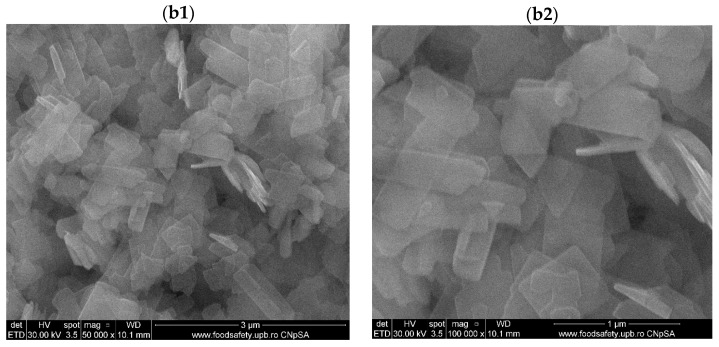
SEM micrographs for (**a**) ZnO (at (**a1**) 100,000× and (**a2**) 200,000× magnification) and (**b**) CuO nanoparticles (at (**b1**) 50,000× and (**b2**) 100,000× magnification).

The nanoparticles were also analyzed using SEM to obtain information on the morphology and particle size. This information is essential for correlating their antimicrobial activity and identifying the correlation with the synthesis parameters (e.g., temperature and pressure). Figure 4 illustrates the SEM micrographs for both ZnO and CuO nanoparticles.

ZnO and CuO nanoparticles have different particle morphologies and sizes based on several factors, even if the same synthesis method was used under the same parameters. Using hydrothermal synthesis, ZnO nanoparticles were obtained with spherical particle morphology and sizes in the nanometric range. They tended to agglomerate; nevertheless, the edges of the spheres were distinct, and there was also uniformity in terms of their morphology and sizes. In contrast, CuO nanoparticles exhibited a platelet morphology, with each micron-sized platelet being oriented in a specific manner, stacked one above the other, resulting in dimensional inhomogeneity among the particles.

**Figure 5:**
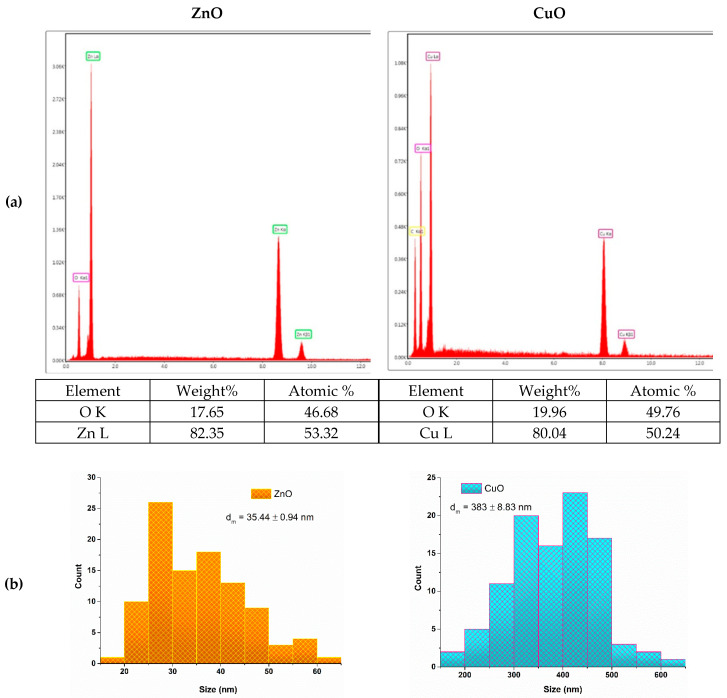
(**a**) EDS results and (**b**) particle size distribution for ZnO and CuO nanoparticles.

The EDS results (Figure 5) reveal the elemental composition of the samples, confirming the presence of zinc and oxygen in the ZnO sample and copper and oxygen in the CuO sample. These findings further validate the successful synthesis of the targeted nanoparticles.

**Figure 6:**
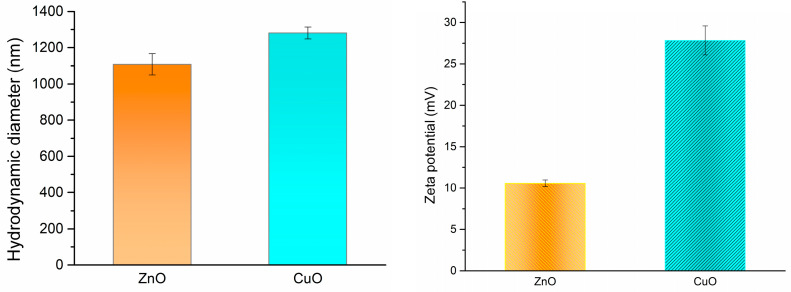
DLS results for ZnO and CuO nanoparticles.

ZnO and CuO powders were analyzed using DLS to assess their hydrodynamic diameter and zeta potential. The findings for both nanoparticle samples are presented in Figure 6.

The hydrodynamic diameter of the nanoparticles reveals only minor differences between the samples, despite their distinct morphology and physical sizes as observed in the SEM micrographs. The smaller size of the zinc oxide nanoparticles is associated with their high tendency to agglomerate, resulting in the formation of nanoparticle clusters. Consequently, when measured using the DLS technique, these clusters show a larger hydrodynamic diameter of 1107.5 nm. In contrast, the physical size of the CuO nanoparticles, along with the hydrodynamic diameter of 1281.2 nm, suggests that these particles remain dispersed. This observation is further supported by the zeta potential values obtained for both samples. The zeta potential of 10.57 mV for ZnO indicates moderate stability, because of its tendency to agglomeration, whereas the zeta potential of 27.83 mV for CuO indicates good stability of the particles in suspension.

**Figure 7:**
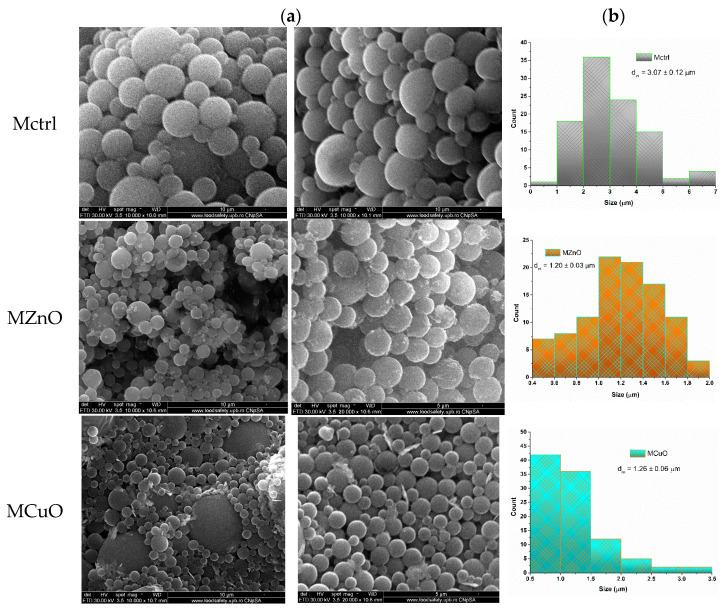
(**a**) SEM micrographs performed at magnification of 10,000× and 20,000×, and (**b**) size distribution of Mctrl, MZnO, and MCuO.

Figure 7 comprises a visual summary of the morphology and size of PLGA-based polymeric microspheres incorporating the two types of antimicrobial nanoparticles. Additionally, a control sample designated as Mctrl was developed to assess the changes occurring during microspheres’ interaction and formation with ZnO and CuO nanoparticles.

The morphology of the microspheres remains consistent even after the incorporation of nanoparticles, exhibiting well-defined contours and maintaining a complete spherical shape. However, the addition of these nanoparticles appears to influence the size of the microspheres. Specifically, the average size of the control sample (Mctrl) is 3.07 µm, whereas MZnO has an average size of 1.20 µm, and MCuO measures 1.26 µm. These data indicate a decrease in the average size of the microspheres with the incorporation of both types of nanoparticles when compared to Mctrl. This reduction can be attributed to the nanoparticles acting as a framework for forming the microspheres, leading to a physical encapsulation process that differs from the control sample, which contains a central void. However, it is observed that in the case of the MCuO sample there are also microspheres of much larger sizes compared to the majority with uniform sizes. Additionally, while the incorporation of nanoparticles appears successful, there is evidence of an excess deposition on the surface of the microspheres.

**Figure 8:**
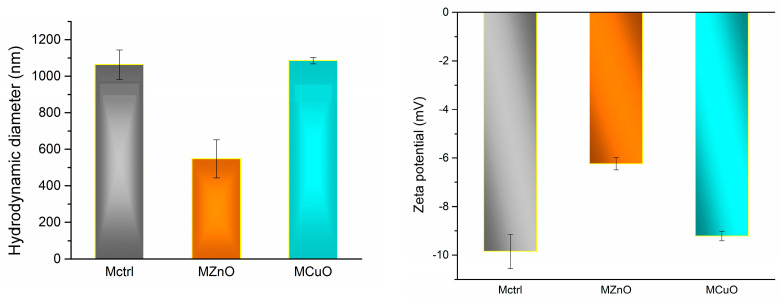
DLS results for Mctrl, MZnO, and MCuO microspheres.

The microspheres were subjected to DLS analysis to assess their size distribution and stability in dispersion. The results are presented in Figure 8.

In terms of hydrodynamic diameter, the microspheres exhibit the following measurements: 1063.63 nm for Mctrl, 547.7 nm for MZnO, and 1085.2 nm for CuO. Notably, the CuO microspheres exhibit nearly double the size of those containing ZnO, indicating a significant difference in particle size. The utilization of an ultrasonic probe during the formation of these microspheres proved beneficial in dispersing the nanoparticles, which, in turn, influenced the hydrodynamic properties of the resulting microspheres. The larger physical dimensions of the CuO particles contribute to the increased hydrodynamic size observed for these microspheres compared to those with ZnO. Furthermore, while the physical dimensions of the microspheres containing nanoparticles, as presented in Figure 6, generally fall within suitable ranges, it is noteworthy that the MCuO sample contains microspheres that display a wider size distribution, with some particles being considerably larger than the majority. In terms of stability, all three samples exhibit moderate stability in liquid media, with zeta potential values of −9.94 mV for Mctrl, −6.23 mV for MZnO, and −9.21 mV for MCuO. The negative charge of all samples is attributed to the presence of PLGA used in the formation of microspheres, due to the deprotonation of carboxyl groups in aqueous environments [48,49].

### 3.2. Characterization of Hydrogel-Type Samples: H_Mctrl, H_MZnO, H_MCuO

The development of collagen-based hydrogels resulted in the creation of three distinct samples. Their characterization will involve comparisons between the control sample, H_Mctrl, which contains no nanoparticles, and the samples H_MZnO and H_MCuO. This analysis aims to elucidate the physicochemical properties of hydrogels and their interactions with eukaryotic and prokaryotic cells.

**Figure 9:**
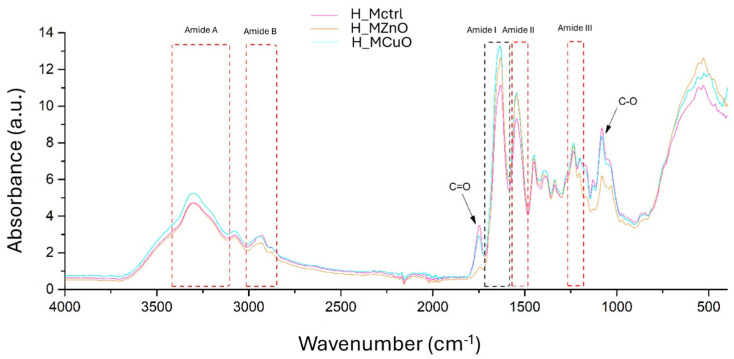
FTIR spectra of H_Mctrl, H_MZnO, and H_MCuO hydrogels.

The first investigation employed FTIR analysis, as illustrated in Figure 9. This analysis aims to identify the functional groups present in the three hydrogels and validate their chemical structures.

All three spectra exhibit the key functional groups characteristic of collagen, which serves as the primary polymeric matrix in which the simple PLGA microspheres, along with zinc oxide and copper oxide nanoparticles, are distributed. Notably, around the wavenumber of 3300 cm^−1^, the Amide A vibration band—indicative of N-H stretching—confirms the presence of the collagen structure. No significant changes in this band are observed after the addition of nanoparticles to the collagen-based formulations, suggesting the preservation of the collagen’s integrity. Additionally, Amide B is present in all three spectra, representing the C-H vibration band. There is a slight variation in band intensity, likely resulting from interactions between the nanoparticles and the PLGA microspheres. Amides I (C=O stretching vibration), II (N-H bending and C-N stretching), and III are also detected, further affirming the collagen structure. Compared to the control sample, these amides exhibit minor differences in position and intensity, which can be attributed to the interactions between the nanoparticles and the scaffold protein. The presence of PLGA is indicated by the C=O group found in the 1750–1800 cm^−1^ region. Furthermore, specific vibrations associated with metal oxides are present in the lower wavenumber region (500–1000 cm^−1^). Both H_MZnO and H_MCuO samples display new bands compared to the H_Mctrl spectrum, signifying the successful integration of the nanoparticles within the collagen matrix.

**Figure 10:**
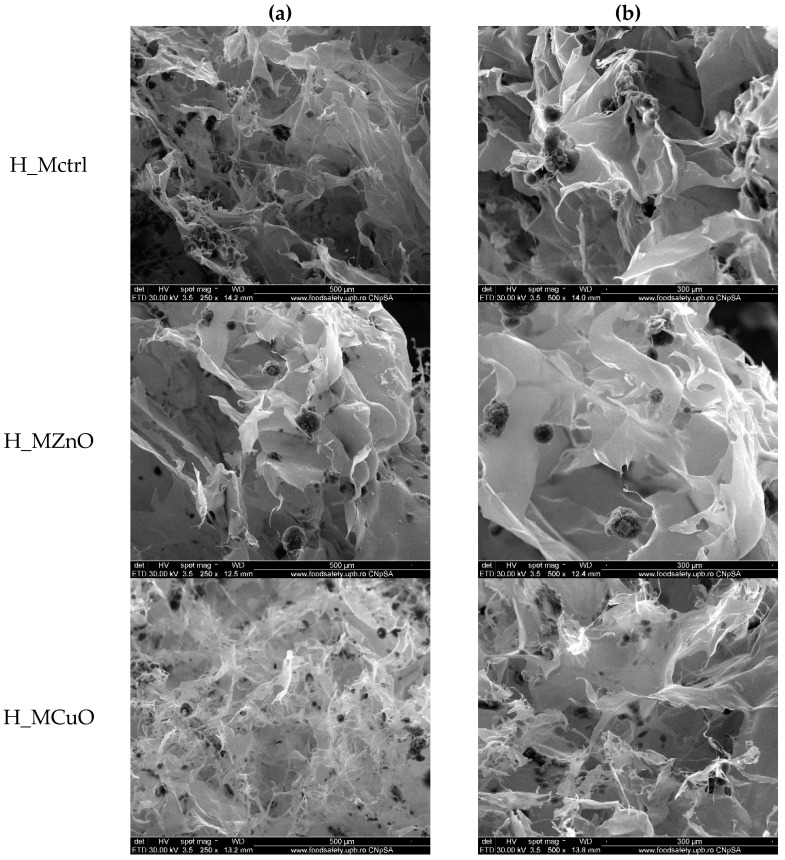
SEM micrographs of H_Mctrl, H_MZnO, and H_MCuO hydrogels, performed at magnifications of (**a**) 250× and (**b**) 500×.

Following the spectroscopic confirmation of the functional groups identified in the composition of the three hydrogels, the next step involves examining their morphology. As such, Figure 10 presents the SEM micrographs of the three hydrogels, performed at magnifications of 250× and 500×.

The representative micrographs of the three hydrogels demonstrate the uniform distribution of polymeric microspheres on the surface and throughout the layers of the collagen scaffold. The hydrogels exhibit a desirable porous structure advantageous for wound healing applications, as these pores facilitate the healing process. Notably, the incorporation of nanoparticle-containing microspheres does not adversely affect the porous architecture of the hydrogels. PLGA microspheres introduced into the collagen matrix tend to form a multilayered arrangement, with larger spheres on the surface and smaller spheres adhering to them.

**Figure 11:**
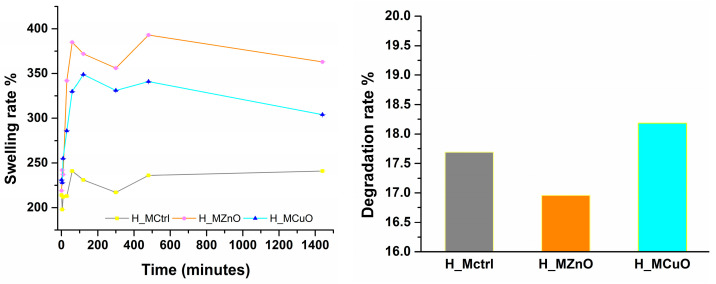
Time-dependent swelling and degradation rate evaluation of H_Mctrl, H_MZnO and H_MCuO hydrogels, after 24 h–1440 min.

Collagen-based hydrogels containing PLGA polymeric microspheres encapsulating ZnO and CuO nanoparticles, respectively, were evaluated for their swelling capacity in contact with PBS over time. Additionally, the degradation of the hydrogels was assessed following the swelling rate evaluation, as this is also a crucial property of the dressings developed in this study. Figure 11 illustrates the swelling rates of the hydrogels over a 24-h (1440 min) period, along with their degradation observed after this time interval.

The results from the swelling rate evaluation of the hydrogels reveal that the hydrogel incorporating zinc oxide nanoparticles (H_MZnO) exhibited the highest liquid retention rate within its structure, followed by the hydrogel with copper oxide nanoparticles (H_MCuO), while the control sample (H_Mctrl) demonstrated the least liquid retention. Notably, all three samples showed promising results for the intended application, as they exhibited high swelling rates from the initial point, with only slight fluctuations observed over the 24-h testing period. However, after this interval, a decline in the swelling rate was noted, particularly for the nanoparticle-containing samples, which correlates with the degradation assessment results. All three samples exhibited maximum degradation rates of up to 18% after 24 h of immersion in PBS, with the highest degradation percentage observed in the H_MCuO sample and the lowest in the H_MZnO sample.

**Figure 12:**
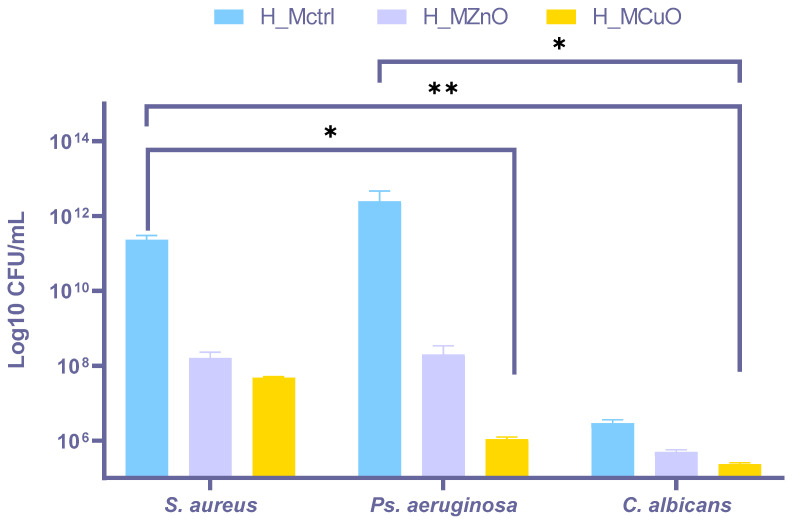
Graphic representation of biofilm modulation of antimicrobial hydrogels—H_Mctrl, H_MZnO, H_MZnO activity after 24 h incubation with *S. aureus*, *Ps. aeruginosa*, and *C. albicans*; * *p* < 0.05; ** *p* < 0.01.

The hydrogels were incubated for 24 h in the presence of two bacterial strains—one Gram-positive (*S. aureus*) and one Gram-negative (*P. aeruginosa*)—and the fungus *Candida albicans* to assess their biofilm inhibition capacity. The results are graphically represented in Figure 12 and compared to the H_Mctrl sample, which displayed no antimicrobial efficacy.

After 24 h of incubation, the results indicate that the nanoparticle-containing hydrogels exhibit significant antimicrobial effects against *S. aureus*, and *Ps. aeruginosa* while limited antifungal activity is observed on *C. albicans.* A distinct antimicrobial response is observed based on the type of nanoparticles used. In tests involving the *S. aureus* strain, the results indicate similar antimicrobial efficacy between H_MZnO and H_MCuO. However, the modulation of the *P. aeruginosa* biofilm reveals that the copper oxide nanoparticles in H_MCuO exhibit a higher antimicrobial efficacy compared to the zinc oxide nanoparticles present in the H_MZnO sample. For *C. albicans*, the values recorded are quite similar to those of the control sample, which lacks antimicrobial agents. Nevertheless, H_MCuO demonstrates notably greater activity in controlling fungal infections.

Considering that the biofilm modulation results are obtained after 24 h of incubation, both hydrogels containing ZnO and CuO demonstrate favorable outcomes. The swift action of the antimicrobial agents against potential wound-related strains highlights their efficacy, which is particularly desirable. Both H_MZnO and H_MCuO hydrogels exhibit antimicrobial activity; however, a comparison reveals a tendency for H_MCuO to offer greater protection against pathogens.

**Figure 13:**
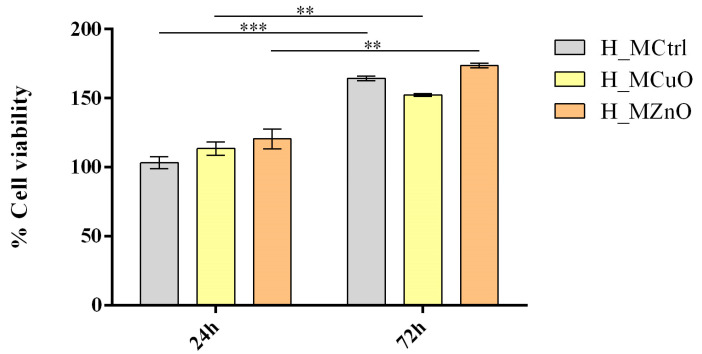
Graphic representation of human keratinocytes viability after 24 h and 72 h of contact with the tested materials (Statistical significance: ** *p* ≤ 0.01; *** *p* ≤ 0.001). All experiments were performed in triplicate, and the results represent the mean of three independent experiments.

To investigate the biocompatibility of the materials with human keratinocytes, different parameters regarding cell health and integrity were assessed. The MTT assay was employed to assess the metabolic activity of cells after contact with the tested materials as a measure of the capacity of the novel materials to sustain cell viability and proliferation (Figure 13). After 24 h, the cell viability of keratinocytes cultured on H_MCuO and H_MZnO was slightly increased compared to the experimental control (H_MCtrl). At 72 h, a statistically significant increase in cell viability compared to 24 h was observed across all conditions, suggesting that all tested materials sustain cell proliferation. The cell viability was similar to the experimental control for both experimental conditions (H_MCuO and H_MZnO), keratinocytes cultured on H_MZnO presented a higher metabolic activity in comparison with H_MCtrl and H_MCuO.

**Figure 14:**
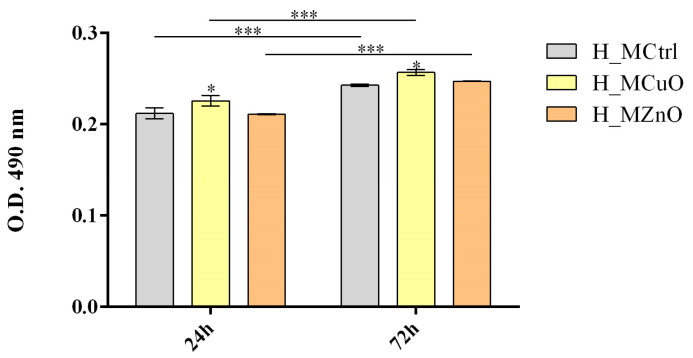
Graphic representation of the LDH levels after 24 h and 72 h of human keratinocytes contact with the tested materials (Statistical significance: * *p* ≤ 0.05; *** *p* ≤ 0.001). All experiments were performed in triplicate, and the results represent the mean of three independent experiments.

Furthermore, the LDH assay was performed to assess cell membrane integrity of keratinocytes after contact with the materials as a measure of the materials cytotoxicity (Figure 14). At 24 h, LDH release was slightly increased in the culture medium of human keratinocytes cultured on H_MCuO as compared with H_MCtrl, suggesting a mild cytotoxic effect. This pattern was maintained after 72 h of culture, indicating that exposure to H_MCuO leads to sustained, but limited, membrane damage over time as compared with H_MCtrl. In contrast, keratinocytes cultured on H_MZnO exhibited LDH levels comparable to those quantified in the H_MCtrl samples at both 24 h and 72 h, suggesting that this material does not compromise membrane integrity and does not induce significant cytotoxic effects. The lack of a substantial increase in LDH release in the H_MZnO group further supports its biocompatibility, reinforcing the findings from the MTT assay that indicated enhanced cell viability and metabolic activity.

**Figure 15:**
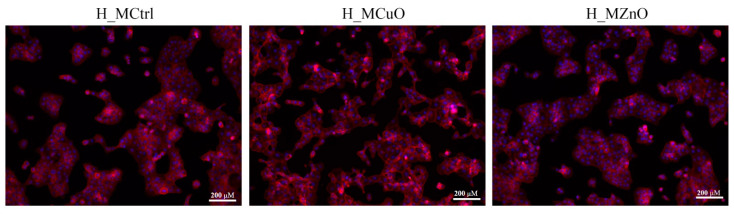
Fluorescence micrographs showing the cytoskeleton of human HaCaT keratinocytes after 24 h of contact with the tested materials. The actin filaments are stained with phalloidin-TRITC (red) and cell nuclei with DAPI (blue).

To further investigate the effects of the tested materials on human keratinocytes, cytoskeletal organization was assessed using phalloidin/DAPI staining after 24 h of culture (Figure 15) to highlight cell morphology, adhesion, and distribution. The HaCat cells cultured on the control surface (H_MCtrl) exhibited a well-organized cytoskeleton with a typical epithelial morphology characterized by a uniform distribution of actin filaments and tight cell–cell interactions. Similarly, on H_MZnO material, the keratinocytes exhibited an epithelial-like morphology, with well-defined actin filaments and cohesive cell clusters, comparable to the experimental control. In contrast, human keratinocytes cultured on H_MCuO showed a more dispersed organization with a looser cytoskeletal structure.

## 4. Discussion

This study aimed to develop and test improved collagen-based hydrogels by adding PLGA polymeric microspheres with distinct content of two types of nanoparticles with antimicrobial properties: zinc oxide and copper oxide. The problem of infections found in chronic wounds is constantly spreading, and current drug therapies can no longer cope due to the development of resistance in bacterial strains. Therefore, using hydrogel-type dressings with controlled release of antimicrobial nanoparticles allows for solving the problem in a new technological manner thanks to the developed formulations involving nanoparticles recognized for their remarkable properties.

The study began by synthesizing zinc oxide and copper oxide nanoparticles using hydrothermal methods. This technique enables precise control over synthesis parameters, such as temperature and pressure, allowing for the customization of particle sizes and morphologies. The resulting powders underwent analysis, beginning with XRD, which confirmed the single-phase composition of zinc oxide in the ZnO sample and copper oxide in the CuO sample, as well as the overall crystallinity of both materials. The FTIR spectra for the ZnO and CuO samples confirm the presence of metal-oxygen groups, which is evident in the 1000–500 cm^−1^ range and characteristic of Zn-O and Cu-O interactions. The synthesis parameters, namely temperature and reaction time, significantly influence the morphology and size of the produced nanoparticles. At a synthesis temperature of 160 °C and a reaction duration of 20 min, predominantly spherical zinc oxide nanoparticles were obtained, with an average particle size of 35.44 ± 0.94 nm.

A study conducted by H.S. Wasley et al. investigated the influence of time and temperature on the physical properties of ZnO nanoparticles synthesized using the same method as in our research. Their findings demonstrated that ZnO nanoparticles exhibit varying sizes and morphologies due to different synthesis durations and temperatures. They performed five experiments at 150 °C, varying the reaction time to 8, 12, 16, 20, and 24 h. Concurrently, they conducted five additional experiments, maintaining a constant reaction time of 12 h while varying the temperature to 100, 125, 150, 175, and 200 °C [50]. Similarly, Sonalika Agarwal et al. synthesized ZnO nanoparticles via the hydrothermal method, setting the temperature at 150 °C for two different reaction times: 17 h and 19 h. They observed the formation of nanoflower morphologies at the shorter reaction time, while at the longer duration, they obtained ZnO nanorods [51]. This indicates that while we used the same synthesis method and reaction temperature, our study yielded different sizes and distinct morphologies influenced by the set reaction time. The same trends were observed for CuO nanoparticles. F. Janene et al. [52] employed hydrothermal synthesis to produce CuO nanoparticles at 180 °C, varying the reaction time to 2, 4, 12, and 24 h. At the shortest reaction time, they obtained platelet-type morphologies similar to those observed in our study. While the same morphology was maintained at a 4-h reaction time, the particles were thicker. However, after 12 h, the morphology shifted to polyhedrons, and at 24 h, this morphology remained but with the addition of other platelets on the surfaces of the particles.

Thus, while the same hydrothermal synthesis parameters were utilized, various other factors influenced the crystallization of the particles differently. As indicated by XRD analysis referencing the PDF-ICDD file with code 04-015-2634, zinc oxide crystallizes in a hexagonal structure. This crystal structure promotes the formation of particles with a spherical morphology. In contrast, copper oxide crystallizes in a monoclinic structure, as specified in the PDF-ICDD database with reference code 00-045-0937, which favors the development of platelet particle morphologies. Additionally, the size of the particles is also affected by the nucleation energy requirements for both ZnO and CuO nanoparticles. In practice, larger particles, such as those formed from CuO, necessitate a higher nucleation energy than ZnO. The crystallization kinetics for ZnO nanoparticles are faster and more uniform, facilitating the formation of spherical nanometric particles. In contrast, CuO exhibits slower nucleation kinetics, which results in larger platelet morphologies [53,54,55,56,57]. This observation is further corroborated by the DLS results obtained for the two nanoparticle powders. The zeta potential values indicate that ZnO nanoparticles exhibit moderate stability, likely due to their small size, which predisposes them to aggregate and destabilize the suspension. In contrast, the CuO nanoparticles demonstrate high stability.

Three types of PLGA microspheres (i.e., control microspheres, ZnO nanoparticle-containing microspheres, and CuO nanoparticle-containing microspheres) were synthesized and analyzed. Using an ultrasonic probe in the PLGA-PVA mixture generates acoustic energy that induces acoustic cavitation, facilitating the formation of bubbles associated with the PLGA-coated spheres [58,59,60]. The control microspheres exhibited micron dimensions of approximately 3 µm, whereas the incorporation of nanoparticles resulted in significantly smaller dimensions, with measurements of 1.20 µm for MZnO and 1.26 µm for MCuO. Thus, the solid particles in the hydrophilic phase can act as nucleation sites around which microspheres form, in contrast to the hollow core observed in the control microspheres (Mctrl) [61,62,63].

The microspheres were incorporated into a collagen matrix to create three distinct hydrogels to address chronic wounds prone to infections. FTIR spectroscopy revealed the presence of key functional groups in collagen, with the vibration bands indicating alterations in position and intensity upon the addition of nanoparticles. This suggests that ZnO and CuO nanoparticles successfully integrated into the collagen matrix. This integration is further demonstrated by SEM analysis of the three hydrogels, which shows that Mctrl, MZnO, and MCuO are uniformly distributed across the entire surface and within the collagen layers while maintaining their spherical morphology. Moreover, due to the hydrophilic nature of collagen, an increase in capillary forces may promote the rearrangement of smaller microspheres on the surfaces of larger ones [64]. A crucial characteristic of an ideal wound dressing is its capacity to absorb exudate and fluids from the wound. To assess this property, the hydrogels were evaluated over time to estimate their swelling and degradation rates in a PBS after 24 h. The results were promising, with the hydrogels exhibiting swelling values exceeding 200% within the first few min of contact with the PBS. Additionally, after 24 h, a degradation process commenced, resulting in up to 19% degradation of the samples, an important factor in the development of effective dressings.

The antimicrobial potential and efficacy of the nanoparticle-containing hydrogels were evaluated against *Staphylococcus aureus*, *Pseudomonas aeruginosa*, and *Candida albicans*, which are among the most prevalent pathogens associated with chronic wounds that are difficult to heal [65,66,67]. The results highlighted notable differences in antimicrobial activity between the two types of nanoparticles depending on the microbial strain. The H_MCuO hydrogel exhibited a more substantial inhibitory effect on biofilm formation, while H_MZnO exhibits lower efficacy, it is still sufficient to demonstrate its well-recognized antimicrobial properties. The results demonstrate that both ZnO and CuO nanoparticle-loaded hydrogels possess antimicrobial activity, with CuO showing greater effectiveness, particularly against *P. aeruginosa*. This enhanced effectiveness can be attributed to CuO increased production of reactive oxygen species (ROS), greater release of Cu^2+^ ions, and stronger interactions with bacterial membranes, which together cause oxidative stress, protein damage, and membrane disruption [68,69]. Although ZnO also exhibits significant antibacterial properties, it appears to be slightly less effective against the Gram-negative bacteria *P. aeruginosa*, potentially due to differences in ROS generation and nanoparticle surface interactions. The limited antifungal activity of both ZnO and CuO against *C. albicans* indicates that the fungal cell walls, which are rich in chitin and ergosterol, create a barrier that reduces metal ion uptake and ROS-induced damage [70,71]. Given the prevalence of *S. aureus* and *P. aeruginosa* in chronic wound infections, CuO-loaded hydrogels may represent a more effective antimicrobial dressing, especially for addressing antibiotic-resistant Gram-negative bacteria [72,73,74,75,76,77,78,79,80,81,82,83,84,85,86,87,88,89].

Multiple parameters related to cell viability, proliferation, cytotoxicity, and morphology were assessed to evaluate the biocompatibility of the novel materials. The combined results demonstrate that both materials (H_MZnO and H_MCuO) are biocompatible with human keratinocytes. H_MZnO provided an especially favorable environment for HaCaT cells, promoting metabolic activity, maintaining cell membrane integrity, and preserving normal cytoskeletal organization.

While H_MCuO also exhibited good biocompatibility, supporting keratinocyte viability and proliferation, it induced a mild cytotoxic effect, as indicated by increased LDH release and minor cytoskeletal structure and organization alterations. These findings suggest that the observed cytotoxicity is minimal and could be mitigated through fine-tuning the CuO-hydrogel formulation. Previous studies have associated CuO with oxidative stress, DNA damage, and apoptosis in keratinocytes, often leading to significant cytotoxicity [90,91]. However, in this study, the effects of H_MCuO were relatively mild and did not compromise overall cell viability or proliferation. Thus, the minor cytotoxic effects observed are not severe enough to hinder the material’s potential application in chronic wound healing, highlighting the importance of further optimization to enhance its safety profile.

## 5. Conclusions

In this study, two types of nanoparticles, ZnO and CuO, recognized for their medical applications, were incorporated into hydrogel dressings designed to address chronic wounds complicated by infections. These nanoparticles were synthesized via a hydrothermal method under identical reaction parameters, and their distinct chemical and physical properties, inherent to the chosen metal, were successfully characterized. To enhance their sustained activity, the nanoparticles were embedded into PLGA polymeric microspheres, which are widely used in biomedical applications for their ability to protect and prolong the efficacy of active agents. Collagen was selected as the primary hydrogel matrix due to its biocompatibility and suitability as a scaffold material, meeting multiple criteria for ideal wound dressings.

The antimicrobial efficacy of the hydrogels was tested against *S. aureus* (Gram-positive), *P. aeruginosa* (Gram-negative), and *C. albicans* (fungal strain). Results revealed that the antimicrobial effects varied depending on the type of microorganism and nanoparticle incorporated.

The biocompatibility assessment confirmed that both H_MCuO and H_MZnO support keratinocyte viability, proliferation, and cytoskeletal organization, with metabolic activity increasing over time. LDH assay results showed minimal cytotoxic effects for both materials, with no significant compromise of membrane integrity. Phalloidin/DAPI staining further demonstrated well-organized cytoskeletal structures and epithelial-like morphology in all conditions. These findings highlight the potential of both materials for biomedical applications, with comparable biocompatibility to the control, making them promising candidates for tissue engineering.

In conclusion, ZnO and CuO nanoparticles’ distinct physicochemical properties and antimicrobial activities provide a strong foundation for their application in advanced wound dressings. These findings underscore the potential of these nanoparticle-enriched collagen hydrogels as versatile and effective therapeutic options for managing chronic wounds complicated by diverse microbial infections.

While this study provides valuable insights into the properties of H_MZnO and H_MCuO hydrogels, certain limitations should be acknowledged. First, the study did not include mechanical property evaluations of the hydrogel membranes, which could provide further insight into their structural stability and potential applicability in wound healing. Also, the antimicrobial evaluation was primarily based on CFU quantification, without zone of inhibition assays, which could offer additional visual confirmation of antimicrobial efficacy. Lastly, a direct comparison between encapsulated and non-encapsulated nanoparticles within the hydrogel matrix was not performed, which could provide further insight into their antimicrobial performance and release kinetics. Future studies should focus on addressing these limitations to further optimize the hydrogel system for biomedical applications.

## Figures and Tables

**Table 1:** The materials used to develop the antimicrobial hydrogels based on every synthesis stage.

Synthesis Stages	Substance	Chemical Formula
ZnO nanoparticles synthesis	Zinc nitrate hexahydrate	Zn(NO_3_)_2_·6H_2_O
	Sodium hydroxide	NaOH
	Methanol	CH_3_OH
	Ultrapure water	H_2_O
CuO nanoparticles synthesis	Copper II nitrate hemipentahydrate	Cu(NO_3_)_2_·2,5H_2_O
	Sodium hydroxide	NaOH
	Ultrapure water	H_2_O
PLGA microspheres formation	Polylactic-co-glycolic acid (PLGA)	C_5_H_8_O_5_
	Polyvinyl alcohol (PVA)	(C_2_H_4_O)_n_
	Chloroform	CHCl_3_
	Ultrapure water	H_2_O
Hydrogels formation	Collagen	C_57_H_91_N_19_O_16_
	Glutaraldehyde	C_5_H_8_O_2_

**Table 2:** All obtained samples are labeled with their designated codes throughout the manuscript.

Obtained Sample	Sample Code
Zinc oxide nanoparticles	ZnO
Copper oxide nanoparticles	CuO
PLGA–microsphere control	Mctrl
PLGA–microsphere-embedded ZnO	MZnO
PLGA–microsphere-embedded CuO	MCuO
Control hydrogel (collagen + Mctrl)	H_Mctrl
ZnO hydrogel (collagen + MZnO)	H_MZnO
CuO hydrogel (collagen +MCuO)	H_MCuO

## Data Availability

The original contributions presented in the study are included in the article material; further inquiries can be directed to the corresponding author.
